# Foraging area fidelity for Kemp's ridleys in the Gulf of Mexico

**DOI:** 10.1002/ece3.594

**Published:** 2013-05-28

**Authors:** Donna J Shaver, Kristen M Hart, Ikuko Fujisaki, Cynthia Rubio, Autumn R Sartain, Jaime Peña, Patrick M Burchfield, Daniel Gomez Gamez, Jaime Ortiz

**Affiliations:** 1 National Park ServicePadre Island National Seashore, Corpus Christi, TX, 78480-1300; 2 U.S. Geological Survey, Southeast Ecological Science CenterDavie, FL, 33314; 3 University of Florida, Ft. Lauderdale Research and Education CenterDavie, FL, 33314; 4 Cherokee Nation Technology Solutions, contracted to U.S. Geological Survey, Southeast Ecological Science CenterDavie, FL, 33314; 5 Gladys Porter ZooBrownsville, TX, 78520

**Keywords:** Kernel density estimation, *Lepidochelys kempii*, satellite tracking, site fidelity, state-space modeling

## Abstract

For many marine species, locations of key foraging areas are not well defined. We used satellite telemetry and switching state-space modeling (SSM) to identify distinct foraging areas used by Kemp's ridley turtles (*Lepidochelys kempii*) tagged after nesting during 1998–2011 at Padre Island National Seashore, Texas, USA (PAIS; *N* = 22), and Rancho Nuevo, Tamaulipas, Mexico (RN; *N* = 9). Overall, turtles traveled a mean distance of 793.1 km (±347.8 SD) to foraging sites, where 24 of 31 turtles showed foraging area fidelity (FAF) over time (*N* = 22 in USA, *N* = 2 in Mexico). Multiple turtles foraged along their migratory route, prior to arrival at their “final” foraging sites. We identified new foraging “hotspots” where adult female Kemp's ridley turtles spent 44% of their time during tracking (i.e., 2641/6009 tracking days in foraging mode). Nearshore Gulf of Mexico waters served as foraging habitat for all turtles tracked in this study; final foraging sites were located in water <68 m deep and a mean distance of 33.2 km (±25.3 SD) from the nearest mainland coast. Distance to release site, distance to mainland shore, annual mean sea surface temperature, bathymetry, and net primary production were significant predictors of sites where turtles spent large numbers of days in foraging mode. Spatial similarity of particular foraging sites selected by different turtles over the 13-year tracking period indicates that these areas represent critical foraging habitat, particularly in waters off Louisiana. Furthermore, the wide distribution of foraging sites indicates that a foraging corridor exists for Kemp's ridleys in the Gulf. Our results highlight the need for further study of environmental and bathymetric components of foraging sites and prey resources contained therein, as well as international cooperation to protect essential at-sea foraging habitats for this imperiled species.

## Introduction

Foraging resources are critical to sustain an individual's survival, and therefore represent an important component of an animal's fitness (Krebs and Davies 1993). Foraging optimality models suggest that animals will select resources of higher quality over those of lower quality, often resulting in optimal use of a patchy environment (MacArthur and Pianka 1966). Despite decades of study on foraging site selection in laboratories (Greenberg 1984) and the terrestrial environment (Bechard 1982), our understanding of the mechanisms of foraging site selection for marine species is limited (Bjorndal 1997). Attempts to confirm foraging behavior for sperm whales, for example, are usually short in duration and few in number due to logistical constraints (see Watwood et al. 2006), and large-scale marine habitat surveys may provide information on abundance but not necessarily confirm foraging behavior (see Davis and Faragion 1998). Only recently have at-sea foraging sites and behavior been delineated for marine megafauna, including sea turtles (Bailey et al. 2008; Shillinger et al. 2010; Hart et al. 2012).

Adults of certain marine species, such as hard-shelled sea turtles, often take up residence at distinct foraging sites after completion of a nesting season. Evidence is emerging in different locales that turtles spend >1 year at these sites (Broderick et al. 2007 [Mediterranean]; Shaver and Rubio 2008 [Gulf of Mexico]; Marcovaldi et al. 2010 [coastal Brazil]) that may represent particular hotspots of high prey abundance. Because sea turtle species are often subjects of satellite-tracking programs, they can be model organisms for studies of foraging site selection in the marine environment.

**Figure 1:**
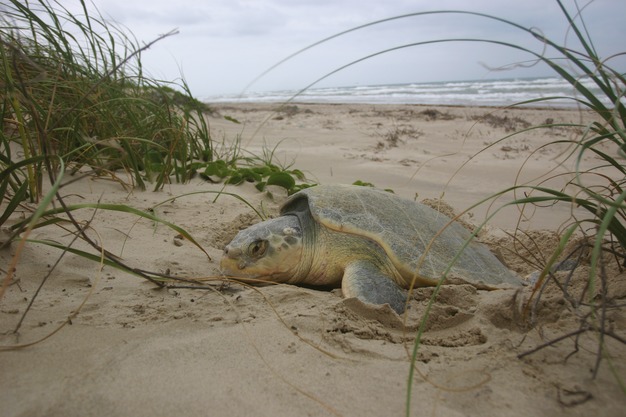
Kemp's ridley turtle is an endangered species that matures at approximately 10–12 years of age. Adults primarily inhabit nearshore waters of the Gulf of Mexico and nest on sandy beaches in Mexico and Texas.

The endangered Kemp's ridley turtle *Lepidochelys kempii* Garman has been the focus of intensive population restoration efforts since the mid-1960s (National Marine Fisheries Service, U.S. Fish and Wildlife Service, and SEMARNAT 2011). Kemp's ridley turtles mature at approximately 10–12 years of age (Fig. 1). Kemp's ridley nesting occurs almost exclusively along the Gulf of Mexico coast, with the largest concentration near Rancho Nuevo (RN), Tamaulipas, Mexico (23.180°N, 97.797°W; Márquez et al. 2005). Since 1978, a bi-national, multi-agency effort has been ongoing to increase Kemp's ridley turtle nesting at Padre Island National Seashore (PAIS), Texas, USA, to form a secondary nesting colony of this native species at this protected beach, as a safeguard against population extinction (Shaver 2005). After decades of intensive conservation and management efforts, increasing numbers of Kemp's ridley nests have been recorded in recent years at nesting beaches in Texas, and Tamaulipas and Veracruz, Mexico (Márquez et al. 2005; Shaver 2005).

Adult Kemp's ridleys utilize habitat primarily within nearshore waters in the Gulf of Mexico (Shaver et al. 2005; Shaver and Rubio 2008) where they forage primarily on Portunid and other crab species (Márquez 1970; Pritchard and Márquez-M 1973; Shaver 1991). Although earlier studies found that RN females were distributed widely along the Gulf coast (Byles 1989; Mysing and Vanselous 1989; Márquez 1994), turtle tracks and tag returns in those studies did not provide enough detail to delineate adult female foraging areas. Core-use foraging areas were delineated for a few adult males netted off RN, but as they remained resident near RN year round (Shaver et al. 2005), these areas are not a good indicator of habitat use by adult females which have been found to be primarily migratory (Shaver and Rubio 2008). Females nesting in Texas comprise the largest component of previously tracked adult Kemp's ridleys, but are only a fraction of the overall adult population; in contrast, the largest nesting aggregation of the species uses beaches in Tamaulipas, Mexico, for reproduction (National Marine Fisheries Service, U.S. Fish and Wildlife Service, and SEMARNAT 2011). Thus, little is known about foraging site selection or locations of foraging areas used by this larger portion of the Kemp's ridley nesting population. Additionally, although core-use areas were recently described for seven adult Kemp's ridleys in the northern Gulf of Mexico (Seney and Landry 2011), all turtles in that study were from a small nesting group on the upper Texas coast, and their core-use foraging areas were clustered in one region. It remains unknown whether a larger percentage of the adult female Kemp's ridley population also uses this particular foraging area, and mechanisms of foraging site selection remain unstudied.

Kemp's ridleys are exposed to many potential threats at sea such as incidental capture in shrimp trawls (Caillouet et al. 1991, 1996; Shaver 1998, 2005), oil spills, dredging operations, and other factors. For sea turtles, adult females represent the segment of the population with the highest reproductive value (RV; Fisher 1930; see Wallace et al. 2008) and as such are extremely important to population growth and recovery. Identifying key foraging areas is a prerequisite to developing management strategies to protect the species in the marine environment in USA and Mexico (Hamann et al. 2010; National Marine Fisheries Service, U.S. Fish and Wildlife Service, and SEMARNAT 2011).

Switching state-space modeling (SSM) has been used to quantify foraging areas for a variety of taxa (Jonsen et al. 2006 [turtles]; Eckert et al. 2008 [turtles]; Bailey et al. 2010 [whales]; Breed et al. 2009 [seals]). SSM was recently used to define foraging areas for female loggerhead turtles *Caretta caretta* Linnaeus from three separate subpopulations in the Gulf of Mexico, USA (Hart et al. 2012), migrations and foraging areas for leatherback turtles *Dermochelys coriacea* Vandelli nesting in the Pacific Ocean (Benson et al. 2011), and internesting movements of olive ridleys *Lepidochelys olivacea* Eschscholtz with respect to marine protected area (MPA) boundaries off central Africa (Maxwell et al. 2011). As a tool, SSM has also been shown to enhance the use of Argos satellite-tracking data for home-range and long distance migration studies of marine animals (Hoenner et al. 2012). In particular, an understanding of where foraging hotspots are, and their frequency of use over time, can be obtained for marine species through the combined use of satellite-tracking and switching SSM (see Jonsen et al. 2003; Eckert al. 2008). Hart et al. (2012) used this approach to delineate foraging areas in the Gulf of Mexico for the loggerhead sea turtle. Here, we use SSM to characterize foraging areas in the Gulf of Mexico for Kemp's ridleys tracked by satellite telemetry after nesting at or near PAIS and RN. Our objectives were to: (1) spatially define foraging areas; (2) define characteristics of foraging areas (i.e., bathymetry, distance from shore, sea surface temperature [SST], and net primary productivity [NPP]) to infer mechanisms of foraging site selection; and (3) examine the consistency of foraging sites selected across years.

## Materials and Methods

### Turtles

Thirty-one platform transmitter terminals (PTTs) were deployed on Kemp's ridleys that nested at PAIS and RN over a 13-year period between 1998 and 2011 (Table S1). At PAIS, 22 PTTs were deployed between 1998 and 2011, and at RN, one PTT was deployed in 2010 and eight were deployed in 2011. Turtles were documented, measured, individually tagged with one passive integrated transponder (PIT) tag and two Inconel flipper tags, and outfitted with PTTs using established protocols (Schmid and Witzell 1997; National Marine Fisheries Service Southeast Fisheries Science Center 2008; Shaver and Rubio 2008). PTTs included models ST-6 (*N* = 2) and ST-20 (*N* = 7) manufactured by Telonics, Inc. (Mesa, Arizona); model KS-101 (*N* = 13) manufactured by Sirtrack (Haverlock North, New Zealand); and models MK-10A (*N* = 1) and MK-10AF (*N* = 8) manufactured by Wildlife Computers (Redmond, WA). PTTs deployed from 1998 to 2007 were programmed with the transmission (duty) cycle of 6 h on/6 h off. In 2008, PTTs were duty cycled on 24 h day^−1^ for the first 106 days and then 6 h on/6 h off thereafter. During 2010 and 2011, KS-101 PTTs were duty cycled 6 h on/6 h off, and MK-10A and MK10AF PTTs were on 24 h day^−1^.

Satellite location data were filtered using Satellite Tracking and Analysis Tool (STAT; Coyne and Godley 2005) available on http://www.seaturtle.org. Location classes (LC) 3, 2, 1, 0, A, and B were used to reconstruct routes and calculate straight line and total distances that the turtles traveled. Argos assigns accuracy estimates of <250 m for LC 3, 250 to <500 m for LC 2, 500 to <1500 m for LC 1, and >1500 m for LC 0 (CLS America 2011). The estimated accuracy remains unknown for LCs A and B. Both traditional least-squares location processing (1998–2010) as well as Kalman filtering (initiated in 2011; Kalman 1960) of location data were performed by Argos. This newly implemented Kalman-filtering algorithm provides more estimated positions and significantly improves position accuracy, most significantly for locations obtained in LCs A and B (Lopez and Malardé 2011).

### Switching SSM and foraging areas

We used switching SSM to characterize the movements of adult nesting Kemp's ridley females in the Gulf of Mexico. Argos satellite locations are recorded at irregular time intervals and are often less precise than published estimates (Vincent et al. 2002) which can be misleading in making inferences even after ad-hoc filtering of outliers (Jonsen et al. 2006). Switching SSM is recommended as the best analytical technique for enhancing Argos tracking data once postprocessed by removing land points and adding back in good Argos locations (Hoenner et al. 2012). Switching SSM has two components accounting for location errors (observation error) and animal behavior (Jonsen et al. (2006; Breed et al. 2009); the observation error is based on the location quality class associated with Argos data. The two-state switching correlated random walk models with the movement process which transits between two behavioral states (see Jonsen et al. 2005 for more detailed model description and Eckert et al. 2008 for equations). Earlier applications defined binary behavioral modes as ‘foraging’ and ‘migration’ (e.g., Breed et al. 2009); however, as we tagged turtles during nesting seasons, we defined the behavioral modes as “foraging and/or nesting” and “migration.” The observation equation translates observed locations to true unobserved locations at equal time intervals.

We specifically used SSM to estimate the date of arrival for each satellite-tagged Kemp's ridley at its foraging destination(s) and the location of foraging sites used prior to arrival at “final” foraging destinations. We summarized data for periods after migration away from nesting beaches, during time periods with “foraging” locations. We assumed that the last foraging locations were the final destination of turtles, unless tracks ended in “migration” locations. We applied a model used in Breed et al. (2009), which is a modified version of a model described in Jonsen et al. (2005) that estimates model parameters by Markov Chain Monte Carlo (MCMC) using WinBUGS via the software program R. We used all tracking data except for LC Z, and we fit the model to tracks of each individual turtle to estimate location and behavioral model every eight hours from two independent and parallel chains of MCMC. Our samples from the posterior distribution were based on 10,000 iterations after a burn-in of 7000 and thinned by five. The convergence was monitored by observing model parameters of two independent chains that were mixed in the trace plots as suggested by Breed et al. (2009). We summarized data for foraging periods until the transmitters stopped sending information or at the time of data synthesis (*N* = 27 until 30 November 2011; *N* = 4 until 7 December 2011).

### Spatial configuration of foraging areas

Using the switching SSM output, we determined the mode associated with each raw data point of the individual turtle tracks. During foraging periods, we considered locations deeper than 100 m to be biologically implausible (see Shaver and Rubio 2008; Seney and Landry 2011) and we filtered these locations out along with any other obviously erroneous locations (on land, spatially very distant, etc.). For foraging periods ≥20 days, we also generated mean daily locations to minimize autocorrelation using the filtered locations; the resulting coordinates provided raw data for kernel density estimation (KDE), a nonparametric method to identify one or more areas of disproportionately heavy use (i.e., core areas; Worton 1987, 1989; White and Garrott 1990). We used the Home-Range Tools for ArcGIS extension (Rodgers et al. 2005) and fixed-kernel least-squares cross-validation smoothing factor (h_*cv*_) for each KDE (Worton 1995; Seaman and Powell 1996). When we observed unequal variance of the *x* and *y* coordinates, we rescaled the data to select the best bandwidth (following Seaman and Powell 1996; Laver and Kelly 2008). Using ArcGIS 9.3 (Environmental Systems Research Institute (ESRI) 2007), we calculated the in-water area (km^2^) within the 50% kernel density contour and to plot the data; in our analysis, a 50% KDE represents a core area of activity at a foraging site (Hooge et al. 2001).

### Foraging area fidelity

We also tested location data for and quantified FAF using the Animal Movement Analysis Extension for ArcView 3.2. Using Monte Carlo Random Walk simulations (100 replicates), we tested tracks during a turtle's time at the foraging ground against randomly generated walks (Hooge et al. 2001). We bounded the range for random walks from −100 to 0 m bathymetry to include only the realistic extent of the in-water habitat. Tracks exhibiting site fidelity indicate movements that are more spatially constrained rather than randomly dispersed. For some animals, we standardized coordinates due to unequal standard deviation of latitude and longitude.

### Characteristics of foraging areas

To characterize at-sea foraging areas selected by individual turtles, we calculated the centroid of each turtle's 50% KDE; if a 50% KDE included multiple activity centers, we calculated the centroid of the largest activity center. We summarized the spatial separation between individual centroids at each foraging ground, and the distance from each centroid to both the nearest land and the mainland. For bathymetry, we used the NOAA National Geophysical Data Center (GEODAS) ETOPO1, 1-arc-minute global relief model of Earth's surface (http://www.ngdc.noaa.gov/m,gg/geodas/geodas.html; accessed 26 January 2012). To depict all foraging locations used by turtles over time, we plotted latitude and longitude of foraging location centroids over time.

We also calculated the number of foraging days in grid cells (25 × 25 km) for 31 individual turtles; the grid extended across the extent of the Gulf of Mexico within the 100-m isobaths. We included all foraging points identified by the SSM except foraging periods less than 2 days. For regression analysis to infer likely environmental correlates of foraging site selection, we used the center of each grid cell to determine: the mean distance to the mainland, distance to the mean tagging/release locations, bathymetry, SST, and NPP. We used Oregon State University ocean productivity data (http://www.science.oregonstate.edu/ocean.productivity/index.php; accessed 15 August 2012) in 2010, in which monthly NPP was derived as a function of chlorophyll, light, and photosynthetic efficiency (Behrenfeld and Falkowski 1997), and 4-km resolution annual SST of Moderate Resolution Imaging Spectroradiometer (MODIS) Aqua Global Level-3 4 μm for 2010 (http://ftp://podaac-ftp.jpl.nasa.gov/OceanTempreature; accessed 15 February 2012). To minimize potential spatial correlation between grid cells, we randomly selected 20% of cells (155 of 778 cells) to analyze the effect of the environmental covariates on turtle days spent in each cell, using a generalized linear model with log transformation in SAS 9.2 GENMOD. We used an alpha level of 0.05 to assess statistical significance.

## Results

### Turtles

Mean size of turtles was 63.4 cm straight carapace length (SCL, ±1.9 SD; Table S1). In a total of 6009 turtle-tracking days, mean individual tracking duration was 193.9 days (± 65.6 SD, range 98–342 days; Table S1). We obtained SSM results for 31 turtles (Fig. S1 and Table S2 provide an example SSM prediction path and the model parameters for turtle 125 – tag 47562). Of these, we successfully tracked 24 turtles to discrete foraging areas to which they displayed site fidelity (i.e., FAF); the remaining seven turtles had foraging locations, but either did not show FAF or did not have enough mean daily locations at a foraging site for kernel density analysis (see below). Five turtles selected >1 foraging site, for a total of 30 estimable KDEs (Tables S3 and S4).

### State-space modeling and foraging areas

**Figure 2:**
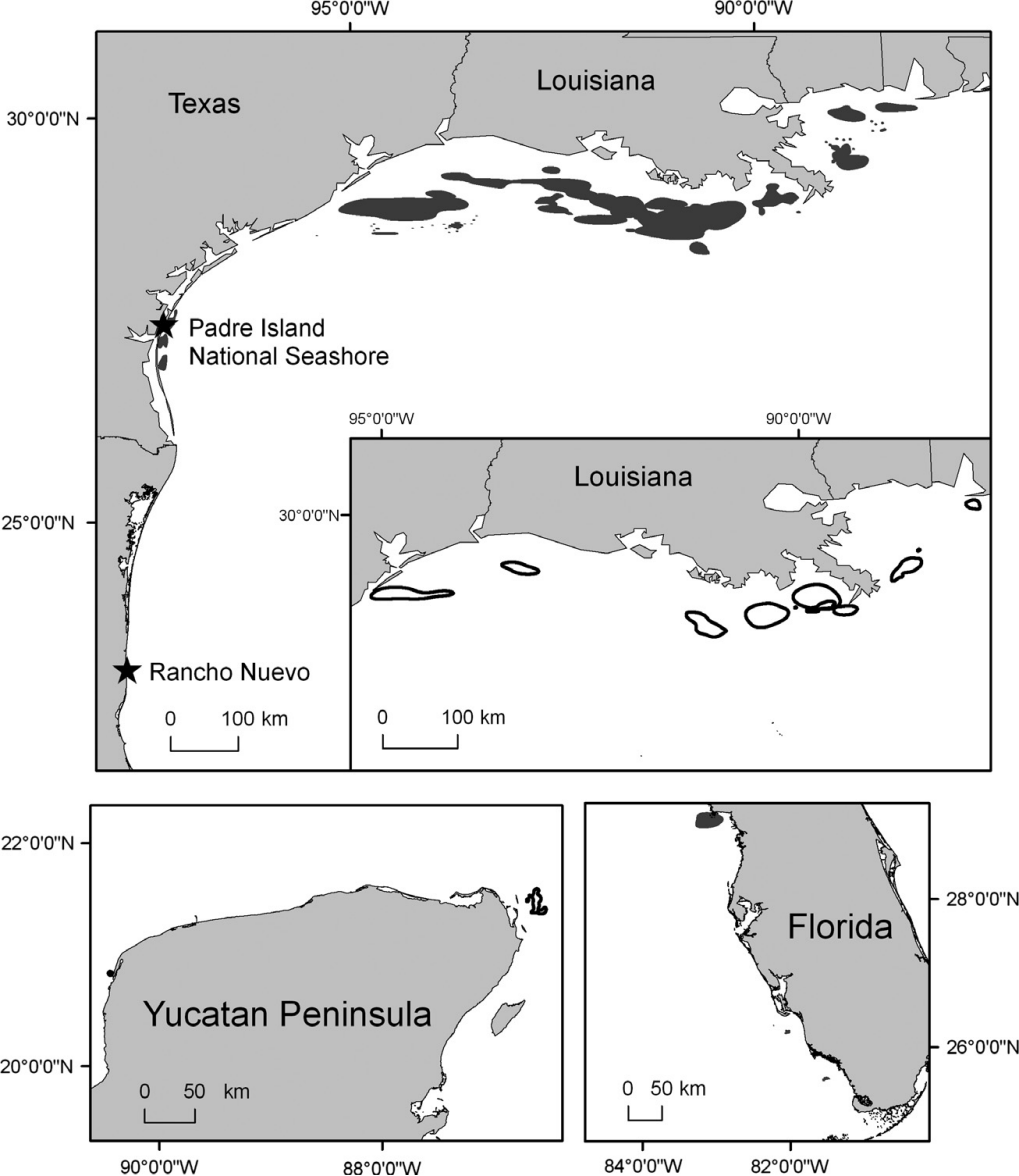
Tagging sites (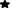) where 31 Kemp's ridleys were intercepted and satellite-tagged after nesting at Padre Island National Seashore (PAIS; TX, USA) and Rancho Nuevo (RN; Mexico). Kernel density estimates (KDEs) for Kemp's ridley turtles in the Gulf of Mexico (*N* = 30); 50% KDEs in shaded gray correspond to turtles originally tagged at PAIS and 50% KDEs in dark outline with no shading correspond to turtles originally tagged at RN.

Turtles traveled a mean distance of 795.0 km (±341.9 SD) to foraging sites. Whereas 22/24 (92%) turtles traveled to and selected foraging sites in waters in the U.S. Gulf of Mexico (USGOM), the other two turtles (8%) traveled to and selected foraging sites in waters in the Mexican Gulf of Mexico (MXGOM; Fig. 2). Foraging sites in the USGOM were selected by all (100%) 19 PAIS turtles and 3/5 (60%) RN turtles. Of the 24 turtles with distinct or discrete foraging areas, 19 turtles selected a single-core foraging area, whereas five turtles selected multiple (i.e., 1–3) foraging sites (mean 1.3 ± 0.7 SD; Table S1), or foraged along their migratory route toward their “final” (F) foraging site destination. However, not all turtles that selected a foraging area selected an F site. For the 18 turtles with distinct F sites, we obtained 25–199 mean daily locations (mean 67.5 ± 40.5 SD; Table S3) at these sites; turtles were resident at F sites for a total period of 1820 days (range 40–263 days; mean 101.1 ± 63.5 SD), which represented a total of 1215 mean daily locations for analysis (Table S3). Furthermore, the size of F sites (i.e., 50% KDEs) ranged from 10.6 to 3877.7 km^2^ (mean 660.8 ± 899.4 SD; Table S3). Bathymetry of F sites ranged from −68.0 m to −1.0 m (mean −7.9 m ± 18.7 SD; Table S3).

For the five turtles with multiple foraging areas, we identified up to three other distinct foraging sites where turtles spent at least 20 days in foraging mode; these sites were utilized by turtles prior to arrival at their F foraging site (Table S4); we also calculated 50% KDE areas for these periods (Table S4). Mean size of F1 foraging sites (i.e., foraging sites used just prior to F sites) was 1314.7 km^2^ (±817.8 SD, *N* = 7); mean size of F2 foraging sites (i.e., foraging sites used just prior to F1 sites) was 1739.0 km^2^ (±1216.8 SD, *N* = 4); and only 1 turtle had an F3 site (i.e., a foraging site used just prior to F2 site) which was 1413.5 km^2^ (see Table S4). Finally, mean sizes of all F1, F2, and F3 sites (50% KDEs) were larger than the mean size of F sites; mean F1 size was 2.0 times as large, mean F2 size was 2.6 times as large, and the single F3 was 2.1 times as large (Table S3).

Overall, we obtained 1896 total mean daily locations for analyses, with 1215 (64%) at F sites, 437 (23%) at F1 sites, 222 (12%) at F2 sites, and 22 (1%) at the single F3 site. Because not every tracking day provided a turtle location, the time period during which turtles were resident at foraging sites differed slightly from the number of mean daily locations. Turtles occupied F1 sites for a total period of 547 days (range 19–207 days), F2 sites for a total period of 251 days (range 21–97 days), and the F3 foraging site for a period of 22 days (Table S4). The total period of tracking days at all foraging sites (F + F1 + F2 + F3), irrespective of KDE limitations, was 2641 days or 44% of the 6009-day tracking period.

### Spatial configuration of foraging sites

All foraging sites were relatively close to land, and in relatively shallow water. Mean distance to the nearest land from centroids at F sites was 25.2 km (±27.0 SD, *N* = 18); mean distance to the mainland coast from the centroids at F sites was 33.2 km (± 25.3 SD; see Table S3). Furthermore, mean bathymetry values (i.e., a proxy for water depths) at F1 centroid locations was −20.4 m (±7.7 SD), −18.5 m (±3.4 SD) at F2 sites, and −19.0 m at the single F3 site; these mean bathymetry values were not significantly different (*F*2,26 = 0.06, *P* = 0.938). Finally, across all foraging sites combined, turtles selected sites a mean 78.4 km (±120.4 SD) from any neighboring turtle's foraging site.

### Foraging area fidelity

**Figure 3:**
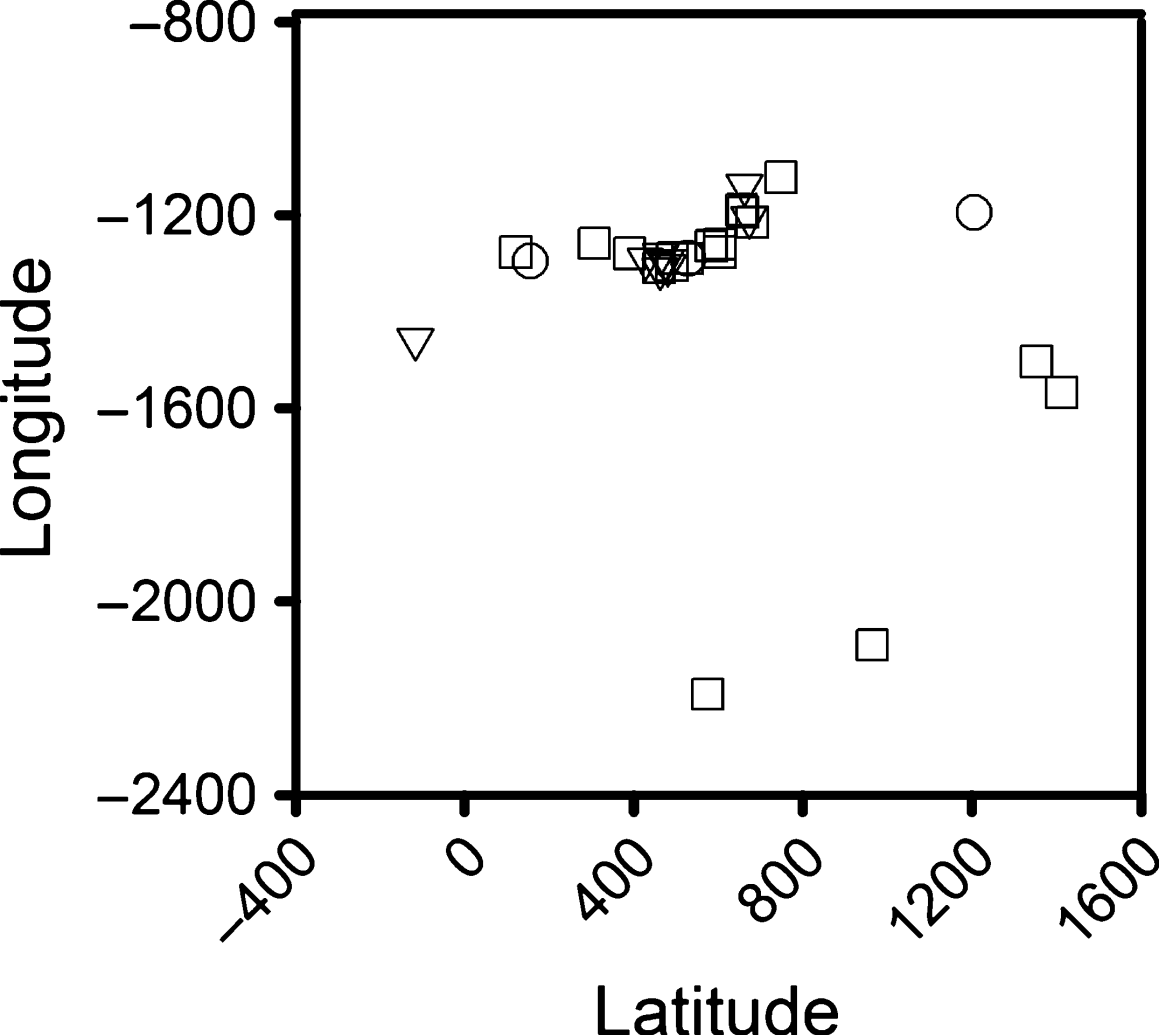
Scatter plot representation of the consistent foraging site selection (in latitude and longitude) for Kemp's ridley turtles over 13 years of tracking. Centroid locations calculated from 50% KDEs were plotted (*N* = 30) in a latitude/longitude scale in thousands with a meter-based projection. Symbols are as follows: circle = 1998–2002; triangle 2003–2007; square 2008–2011.

Turtles often showed fidelity to discrete foraging areas. For all turtles for which we delineated a foraging area, we observed FAF and the proportion of constrained movement paths with higher mean squared distances (MSD) values than observed paths >96.0 (Table 1). Moreover, we observed consistent use of foraging areas over the 13-year tracking period (Fig. 3). We tracked 11 additional turtles (120, 54, 12, RN07, RN08, RN10, RN12, RN15, 319, 145, and 326) to six F sites and five F1 sites, at which FAF was not demonstrated. Mean size of these sites was 1064.2 km^2^ (±861.1 SD; Table S5). However, because turtles at these areas failed the site fidelity test, we did not include the 457 mean daily locations (covering 767 days of tracking) in our previous summaries.

**Table 1:** Foraging area fidelity for Kemp's ridleys at final (F) foraging sites in the Gulf of Mexico as well as F1 (foraging site used prior to F), F2 (foraging site used prior to F1), and F3 (foraging site used prior to F2) sites

Turtle ID	No. foraging sites selected	F	F1	F2	F3
8	1	*P* > 99.0	NA	NA	NA
22	2	NA	*P* > 99.0	*P* > 98.0	NA
54	2	NA	*P* > 99.0	*P* > 99.0	NA
21	1	NA	*P* > 99.0	NA	NA
28	1	*P* > 98.0	NA	NA	NA
30	1	*P* > 96.0	NA	NA	NA
12	1	–	*P* > 99.0	NA	NA
84	1	*P* > 99.0	NA	NA	NA
109	1	*P* > 99.0	NA	NA	NA
33	1	*P* > 99.0	NA	NA	NA
120	1	*P* > 99.0	NA	NA	NA
92	1	*P* > 99.0	NA	NA	NA
125	2	*P* > 98.0	*P* > 99.0	NA	NA
319	2	NA	NA	*P* > 99.0	*P* > 100.0
164	1	NA	*P* > 97.0	NA	NA
321	1	*P* > 99.0	NA	NA	NA
172	1	*P* > 99.0	NA	NA	NA
45	1	*P* > 99.0	NA	NA	NA
230	1	*P* > 99.0	NA	NA	NA
RN04	1	*P* > 99.0	NA	NA	NA
RN09	1	*P* > 99.0	NA	NA	NA
RN11	1	*P* > 99.0	NA	NA	NA
RN12	1	*P* > 99.0	NA	NA	NA
RN13	3	*P* > 99.0	*P* > 98.0	*P* > 99.0	NA

NA, not available.

### Characteristics of foraging sites

**Figure 4:**
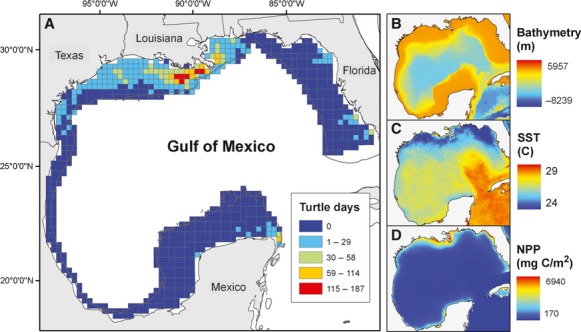
(A) Foraging habitat and environmental characteristics of foraging sites selected for *N* = 31 female Kemp's ridley turtles from 1998 to 2011. The grid is divided into 25 × 25 km cells, with 100-m isobaths as a bounding layer. (B) Bathymetry coverage; (C) SST coverage; (D) NPP coverage.

Over all foraging areas, mean NPP was 3625.4 mg C/m^2^/day (±1131.7 SD) and mean SST was 25.1°C (±0.9 SD) within an overall narrow SST range 24.1–27.6°C. (Table S6). Furthermore, we found consistently high numbers of turtle foraging days in specific grid cells (see “warmer”-colored cells in Fig. 4, all turtles combined). Regression analysis indicated that all factors (i.e., bathymetry, distance to shore, distance to release site, NPP, and SST) were significant predictors of high-use foraging sites (Table 2, see also Fig. S2).

**Table 2:** Estimated parameters and *P*-values for effects of environmental variables on turtle foraging days per grid cell using generalized linear model with log transformation

Parameter	Estimate	SE	Lower CI	Upper CI	Chi-square	*P*-value
Intercept	20.2033	1.3187	17.6188	22.7878	234.74	<0.0001
Distance to release site (km)	−0.0008	0.0001	−0.0010	0.0005	46.16	<0.0001
Distance to mainland shore (km)	0.0060	0.0014	0.0033	0.0086	19.45	<0.0001
Annual mean SST (°C)	−0.8138	0.0531	−0.9179	−0.7097	234.79	<0.0001
Bathymetry	−0.0026	0.0007	−0.0040	−0.0013	14.48	<0.0001
Mean NPP (mg C/m^2^)	0.0009	<0.0001	0.0008	0.0010	714.45	<0.0001

## Discussion

Through analysis of the largest and longest-term satellite-tracking data set for the species, we demonstrated the importance of nearshore Gulf of Mexico waters as foraging habitat for postnesting Kemp's ridleys; we suggest that critical foraging habitat exists for the species in the northern Gulf of Mexico, particularly off Louisiana, to which turtles show fidelity over time. Furthermore, concentration of core-use foraging areas for turtles tracked from both RN and PAIS supports our assertion and underscores the importance of this habitat for these imperiled marine turtles.

Foraging areas for all PAIS turtles were in USGOM waters. This result is similar to Kemp's ridleys tracked from nesting locations on the upper Texas coast (Seney and Landry 2008), with mean size of foraging areas for six turtles larger than that of F sites for PAIS turtles (see Table S3), but similar to the size of F3 and F1 sites defined here. Thus, there seems to be a consistent mean size of foraging areas across turtles and sites. Contrasting the PAIS turtles, F sites for RN turtles were located in Gulf waters off both the USA (*N* = 3; primarily off Louisiana) and Mexico (*N* = 2). Both turtles in MXGOM waters selected foraging areas off the Yucatan Peninsula; this is the first time core-use foraging areas have been identified for females of this species in MXGOM waters.

In our comparison of foraging site locations across years, we discovered consistent use of distinct foraging areas in the Gulf of Mexico (Fig. 3); turtle use of distinct sites is also supported by recent work (Seney and Landry 2011). Thus, as previously shown for loggerheads in the Gulf of Mexico (Hart et al. 2012), we found that there are consistent and distinct “hotspots” for Kemp's ridley foraging areas in the Gulf of Mexico to which turtles display FAF. These hotspots or high concentration of core-use KDEs clearly exists in the northern Gulf USA waters, as well as off the Yucatan Peninsula, and are therefore important areas for targeting future spatially oriented conservation efforts. Thus, additional tracking of adult females from both the PAIS and RN nesting beaches, coupled with SSM, would help to define other possible foraging hotspots and identify whether their location, size, and temporal use vary annually and with respect to environmental conditions and availability of prey resources.

Using SSM, we also identified and delineated other foraging sites (*N* = 12) used by adult female Kemp's ridleys prior to their F site; these sites were ∼2 times larger overall in area than F sites, but situated similarly with respect to depth and distance to shore. We suspect that the same oceanographic characteristics may exist at these sites compared with the F sites, however, turtles moved on from those sites even if seemingly adequate foraging conditions existed.

Use of SSM allowed for identification of two distinct types of migratory foraging behavior: (1) core use, and (2) alternation between foraging and migration, the latter which may be indicative of “opportunistic” foraging (Shaver 1991). Our results show the first evidence of foraging behavior during migration and suggest that a foraging corridor exists for the species in Gulf of Mexico waters offshore in both USA and Mexico. The existence of F3, F2, F1, and F sites and foraging behavior during postnesting migrations has not previously been documented for any Kemp's ridley turtles.

For leatherbacks in the Pacific Ocean, SSM indicated that the foraging phase was more prolonged and widely dispersed, suggesting that food patches could be less predictable (Bailey et al. 2008). This result is similar to observed behavior for some Kemp's ridleys that used multiple foraging sites; presumably locations of these foraging sites coincide with locations of adequate prey resources, although this hypothesis remains untested. Notably, loggerheads in Gulf waters prey on similar resources used by Kemp's ridleys, and lack alternation between “foraging” and “migration” as seen in the Kemp's ridley turtles (Hart et al. 2012). Perhaps prey resources are not limiting loggerhead behavior, or the two species may be competing for prey in some areas, with the smaller Kemp's ridleys being displaced by the larger loggerheads or with increasing competition from the growing Kemp's ridley population. Regardless, direct, in-water exploration of foraging sites is needed to identify bottom type and confirm availability of prey resources at these biologically important sites.

Many pelagic predators use biological and physical oceanographic features as cues to identify areas of high productivity, foraging in currents or along continental shelves (Suryan et al. 2006). High-use foraging areas in our study were all relatively nearshore (mean 25.2 km) in shallow water (<68 m deep), within a narrow temperature range (SST range 24.1–27.6°C at foraging sites), and in areas of relatively high NPP. However, the majority of foraging sites we identified were in water slightly deeper than that previously reported for Kemp's ridleys at other presumed foraging sites (see Seney and Landry 2011). Unlike loggerheads that will travel to foraging sites over deep water in the Gulf (see Hart et al. 2012), all of the turtles tracked here remained in shallow nearshore habitats; this habitat preference heightens the potential impact human activities in this area will have for the success of the species. Our analysis highlights several environmental characteristics that may make habitats particularly suitable as foraging sites for Kemp's ridleys. However, future research is needed to identify bathymetric features and benthic composition of sites known to be used by Kemp's ridleys for foraging.

Our delineation of distinct foraging zones is paramount for understanding at-sea foraging habitat site selection and therefore, areas of potential conservation concern. Sea turtles in nearshore Gulf of Mexico waters are exposed to incidental capture in shrimp trawls, oil spills, dredging, hypoxia, and other threats. The concentration of Kemp's ridley foraging areas along the coasts of Louisiana, Mississippi, and Alabama is significant as these areas are known for heavy fishing effort and oil production. Recent, unusually high mortality rates for bottlenose dolphins, for example, were recorded along these coasts in 2011 and linked to the combination of cold water and the Deepwater Horizon Oil Spill (Carmichael et al. 2012). Although habitat characteristics and suitability for sea turtles in this region are poorly understood, locations of core-use foraging areas identified here indicate that important habitat exists for Kemp's ridley turtles at these same impacted sites. Whether such foraging sites previously used by Kemp's ridleys will continue to be used with equal frequency in the future, or alternatively abandoned, remains to be seen; it is possible that environmental conditions and prey resources at some of these sites have been altered by the large-scale perturbation of the northern Gulf Deepwater Horizon Oil Spill (Campagna et al. 2011). Additionally, narrow habitat suitability increases the likelihood for extinction (Grinnell 1917; Chase and Leibold 2003) and this is exacerbated by the high level of human impacts in coastal areas.

## Conclusions

Nearshore Gulf of Mexico waters serve as prime foraging habitat for postnesting Kemp's ridley turtles. Our results define critical foraging area hotspots for this species in the northern Gulf of Mexico. Consistent selection of this region by turtles tracked from PAIS over a 13-year period, concentration of core-use foraging areas for turtles tracked from both RN and PAIS, and high FAF underscore the importance of this habitat across time, and for individuals from the largest segment of the nesting population (i.e., RN females). The dispersion of foraging sites indicates that a foraging corridor exists in nearshore Gulf of Mexico waters and underscores the need for international cooperation for conservation of this imperiled species. Additional and continued tracking of adult females from both PAIS and RN nesting beaches is warranted to further delineate this corridor and understand details of turtle behavior linked to foraging site selection, both along the migratory pathway and at ‘final’ foraging sites.

## Supporting Information

**Figure S1.** An example of raw data (open gray circles and gray path) and predicted movement trajectory and behavioral mode of a Kemp's ridley (Turtle ID # 125, tag 47562) tagged in Padre Island National Seashore.

**Figure S2.** Predicted turtle days using estimated coefficients derived using a generalized linear model with log transformation. The grid is divided into 25 × 25 km cells, with 100-m isobaths as a bounding layer.

**Table S1.** Tracking details for satellite-tracked Kemp's ridley turtles.

**Table S2.** An example of posterior of switching state-space model parameters for a satellite-tracked Kemp's ridley turtle.

**Table S3.** Final foraging site characterization for satellite-tracked Kemp's ridley turtles.

**Table S4.** Additional foraging site characteristics for satellite-tracked Kemp's ridley turtles.

**Table S5.** Satellite-tracked Kemp's ridley foraging site characteristics for foraging periods that failed site fidelity tests.

**Table S6**. Net primary productivity (NPP) and sea surface temperature (SST) values in foraging habitat selected by Kemp's ridley turtles.
