# Supplementary material for: Foraging area fidelity for Kemp's ridleys in the Gulf of Mexico
Source: Ecol Evol. 2013 May 28;3(7):2002–12. doi: 10.1002/ece3.594 (PMC3728941; doi:10.1002/ece3.594)
Supplement: Supplementary file 1 [file ece30003-2002-SD1.docx]

SUPPLEMENTAL FIGURES
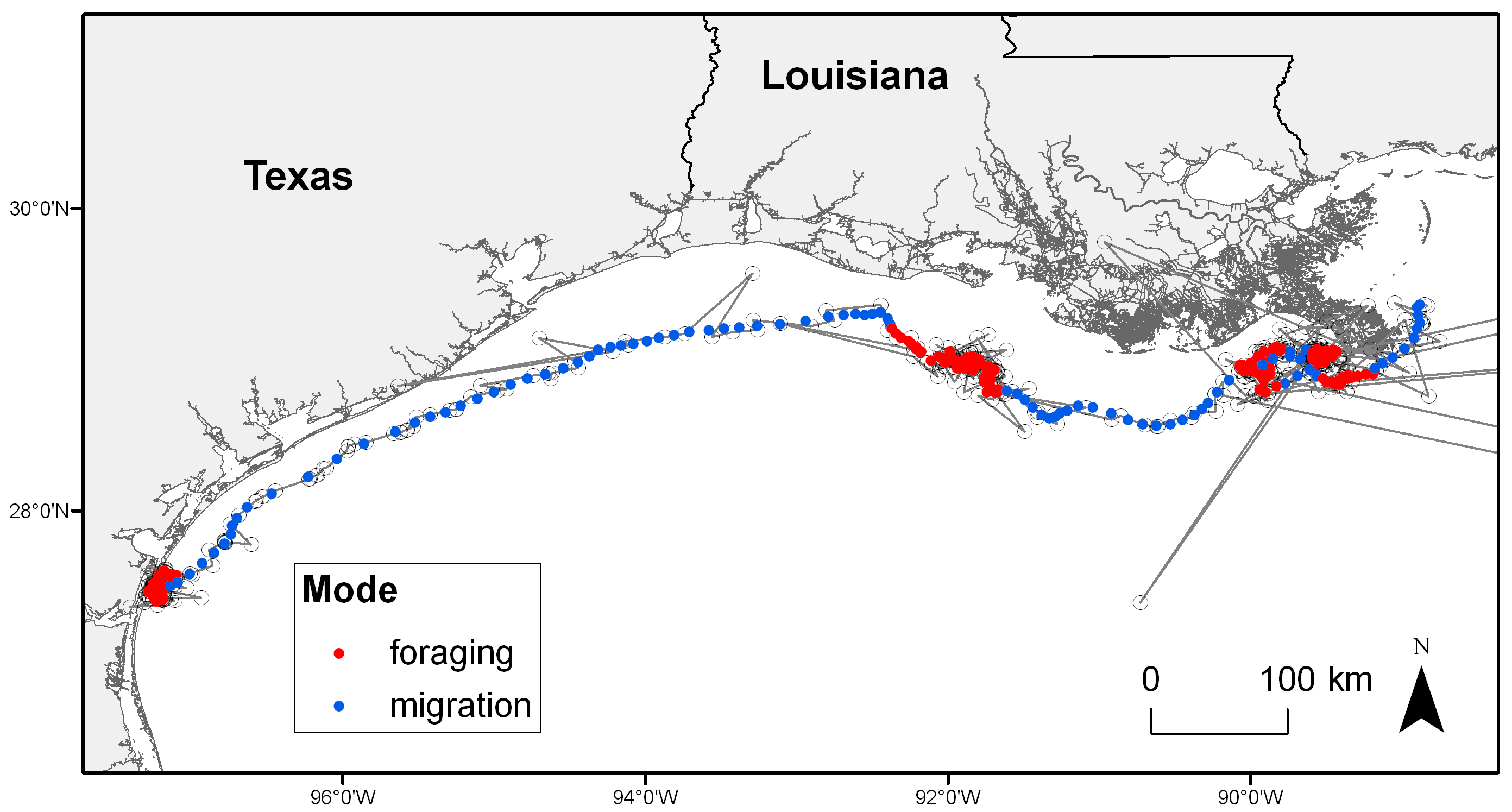


**Fig S1.** An example of raw data (open gray circles and gray path) and predicted movement trajectory and behavioral mode of a Kemps ridley (Turtle ID # 125, tag 47562) tagged in Padre Island National Seashore.


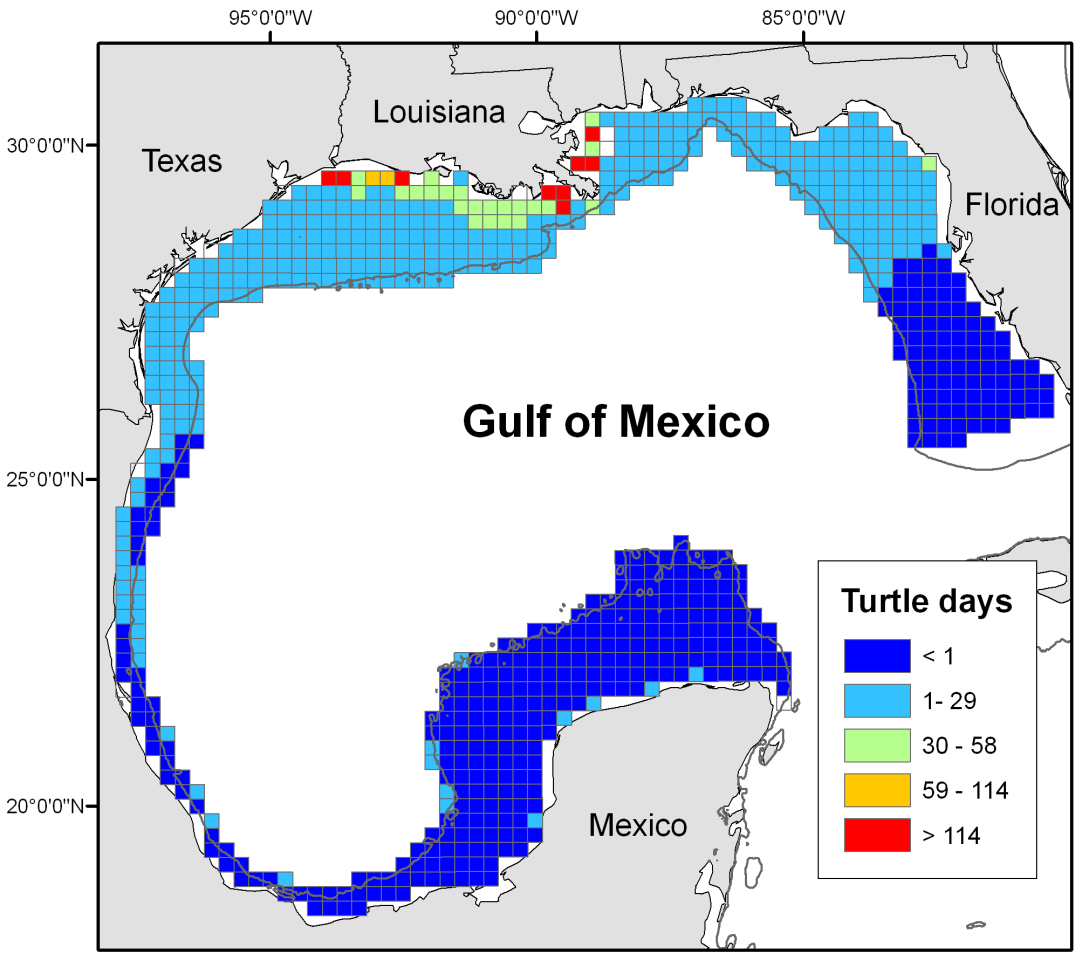


**Fig S2.** Predicted turtle days using estimated coefficients derived using a generalized linear model with log transformation. The grid is divided into 25 x 25 km cells, with 100 m isobaths as a bounding layer.
