# Supplementary material for: Foraging area fidelity for Kemp's ridleys in the Gulf of Mexico
Source: Ecol Evol. 2013 May 28;3(7):2002–12. doi: 10.1002/ece3.594 (PMC3728941; doi:10.1002/ece3.594)
Supplement: Supplementary file 2 [file ece30003-2002-SD2.docx]

SUPPLEMENTAL TABLES

**Table S1.** Tracking details for 31 Kemp's ridleys tracked from nesting beaches in Texas and Mexico, 1998-2011 with straight carapace length (SCL). Turtles tagged at Padre Island National Seashore, Texas, USA are numbered, whereas those tagged at Rancho Nuevo, Mexico are denoted RN04-RN15

| Turtle ID # | Tracking period | No. tracking days | Tag Type | SCL (cm) |
| --- | --- | --- | --- | --- |
| 8 | 4/25/1998 - 12/8/1998 | 227 | ST-6 | 62.9 |
| 22 | 4/13/2000 - 10/14/2000 | 184 | ST-6 | 63.1 |
| 54 | 5/1/2004 - 3/30/2005 | 333 | ST-20 | 64.2 |
| 21 | 5/1/2004 - 9/25/2004 | 147 | ST-20 | 61.3 |
| 28 | 5/8/2005 - 9/23/2005 | 138 | ST-20 | 63.8 |
| 30 | 4/27/2006 - 4/4/2007 | 342 | ST-20 | 62.1 |
| 12 | 4/27/2006 - 3/26/2007 | 333 | ST-20 | 61.3 |
| 84 | 4/27/2006 - 8/3/2006 | 98 | ST-20 | 60.8 |
| 109 | 5/1/2007 - 10/3/2007 | 155 | ST-20 | 63.7 |
| 33 | 4/24/2008 - 9/2/2008 | 131 | KS101 | 67.0 |
| 120 | 4/27/2010 - 8/29/2010 | 124 | KS101 | 64.1 |
| 92 | 6/14/2010 - 10/19/2010 | 127 | KS101 | 65.7 |
| 125 | 5/6/2010 - 10/7/2010 | 154 | KS101 | 63.6 |
| 319 | 4/7/2011 - 10/11/2011 | 187 | KS101 | 59.8 |
| 164 | 4/16/2011 - 11/30/2011* | 228 | KS101 | 63.7 |
| 321 | 4/22/2011 - 11/30/2011* | 222 | KS101 | 62.3 |
| 172 | 4/23/2011 - 11/30/2011† | 221 | KS101 | 64.8 |
| 45 | 4/26/2011 - 11/30/2011† | 218 | KS101 | 64.5 |
| 322 | 4/25/2011 - 9/8/2011 | 136 | MK10AF | 61.7 |
| 145 | 4/23/2011 – 8/2/2011 | 101 | MK10AF | 62.5 |
| 326 | 4/28/2011 - 10/17/2011 | 172 | MK10AF | 60.3 |
| 230 | 4/28/2011 - 9/29/2011 | 154 | MK10AF | 61.4 |
| RN04 | 7/23/2010 - 6/10/2011 | 322 | MK10A | 66.7 |
| RN07 | 4/28/2011 - 11/30/2011* | 216 | MK10AF | 62.6 |
| RN08 | 4/28/2011 - 11/30/2011* | 216 | MK10AF | 62.0 |
| RN09 | 4/28/2011 - 11/9/2011 | 195 | MK10AF | 63.5 |
| RN10 | 4/28/2011 - 11/30/2011* | 216 | MK10AF | 65.2 |
| RN11 | 6/5/2011 - 11/30/2011* | 178 | KS101 | 66.0 |
| RN12 | 6/5/2011 - 11/30/2011† | 178 | KS101 | 63.6 |
| RN13 | 6/5/2011 - 11/30/2011† | 178 | KS101 | 67.0 |
| RN15 | 6/5/2011 - 11/30/2011* | 178 | KS101 | 63.5 |
|  |  |  |  |  |
| *transmitted beyond 11/30/2011 cutoff date for analysis  †data included until 12/7/2011 as turtles remained at final foraging sites | | | | |
|  |  |  |  |  |

**Table S2.** An example of posterior of switching state space model parameters for a Kemps ridley (Turtle ID # 125, tag 47562) tagged in Padre Island National Seashore.

| **Node** | **Mean** | **SD** | **2.5% CI** | **97.5% CI** |
| --- | --- | --- | --- | --- |
| Process uncertainty from mode 1 to 1 (σ1,1) | 0.0042 | 2.62E-04 | 0.0037 | 0.00473 |
| Process uncertainty from mode 1 to 2 (σ1,2) | 4.61E-04 | 1.16E-04 | 2.50E-04 | 6.92E-04 |
| Process uncertainty from mode 2 to 1 (σ1,2) | 4.61E-04 | 1.16E-04 | 2.50E-04 | 6.92E-04 |
| Process uncertainty from model 2 to 2 (σ2,2) | 0.0026 | 1.24E-04 | 0.0023 | 0.002796 |
| Probability of being mode 1 (α1) | 0.9905 | 0.0021 | 0.9860 | 0.9939 |
| Probability of being mode 2 (α2) | 0.0254 | 0.0047 | 0.0168 | 0.0340 |
| Moving speed persistence in mode 1 (γ1) | 0.6726 | 0.0309 | 0.6173 | 0.7629 |
| Moving speed persistence in mode 2 (γ2) | 0.6754 | 0.019 | 0.6357 | 0.7159 |

**Table S3.** Final foraging site characterizations for 24 Kemp's ridley turtles satellite tracked in the Gulf of Mexico between 1998-2011to northern Gulf of Mexico (NGOM), southern Gulf of Mexico (SGOM), western FL (W FL), southwest Florida (SW FL) and Mexico (MX). NA = not available.

| Turtle ID | Foraging site location | Single (S) or multiple foraging (M) sites | Size of 50% KDE (km^2^) | No. mean daily locations | Dates at F (No. days) | | KDE Bandwidth (h_cv_) | Distance to nearest shore (km) | | Distance to mainland (km) | | Bathymetry (m) | |  |  |
| --- | --- | --- | --- | --- | --- | --- | --- | --- | --- | --- | --- | --- | --- | --- | --- |
| 8 | W FL | S | 655.8 | 25 | 7/12/98-12/8/98 (149) | | 0.463 | 5.4 | | 17.6 | | -5 | |  |  |
| 22 | NGOM | M | NA | NA | NA | | NA | NA | | NA | | NA | |  |  |
| 54 | NGOM | M | NA | NA | NA | | NA | NA | | NA | | NA | |  |  |
| 21 | NGOM | S | NA | NA | NA | | NA | NA | | NA | | NA | |  |  |
| 28 | NGOM | S | 3877.7 | 75 | 7/6/05-9/23/05 (79) | | 0.323 | 39 | | 48.4 | | -14 | |  |  |
| 30 | NGOM | S | 1561.4 | 199 | 7/27/06-4/4/07 (251) | | 0.089 | 18.9 | | 30 | | -3 | |  |  |
| 12 | NGOM | S | NA | NA | NA | | NA | NA | | NA | | NA | |  |  |
| 84 | NGOM | S | 472.3 | 38 | 6/9/06-7/29/06 (50) | | 0.213 | 2 | | 3 | | -14 | |  |  |
| 109 | NGOM | S | 1046.4 | 73 | 7/9/07-10/3/07 (86) | | 0.268 | 0.6 | | 35.2 | | -1 | |  |  |
| 33 | NGOM | S | 594.1 | 56 | 7/7/08-9/2/08 (57) | | 0.34 | 37.1 | | 45 | | -17 | |  |  |
| 120 | NGOM | S | 745.18 | 45 | 7/16/10-8/29/10 (44) | | 0.347 | 22.7 | | 30.5 | | -16 | |  |  |
| 92 | SW FL | S | 22.2 | 39 | 9/9/10-10/19/10 (40) | | 0.117 | 21.9 | | 21.6 | | -14 | |  |  |
| 125 | NGOM | M | 179.2 | 75 | 7/14/10-10/7/10 (85) | | 0.119 | 15.1 | | 15.8 | | -14 | |  |  |
| 319 | NGOM | M | NA | NA | NA | | NA | NA | | NA | | NA | |  |  |
| 164 | NGOM | S | NA | NA | NA | | NA | NA | | NA | | NA | |  |  |
| 321 | NGOM | S | 309.7 | 76 | 7/1/11-9/17/11 (78) | | 0.261 | 9.2 | | 36 | | -7 | |  |  |
| 172 | NGOM | S | 860.9 | 102 | 8/16/11-12/7/11* (113) | | 0.312 | 32.1 | | 32.1 | | -41 | |  |  |
| 45 | SW FL | S | 27.2 | 45 | 8/6/11-12/7/11* (123) | | 0.228 | 21.5 | | 27.9 | | -7 | |  |  |
| 230 | NGOM | S | 193.2 | 28 | 7/7/11-9/29/11 (84) | | 0.107 | 2.3 | | 37.2 | | -2 | |  |  |
| RN04 | MX | S | 167.3 | 86 | 9/20/10-6/10/11 (263) | | 0.209 | 0.3 | | 22.2 | | -32 | |  |  |
| RN09 | NGOM | S | 151.3 | 32 | 8/8/11-11/9/11 (93) | | 0.521 | 11.1 | | 11.1 | | -15 | |  |  |
| RN11 | MX | S | 10.6 | 45 | 8/4/11-9/17/11 (44) | | 0.44 | 2.5 | | 1.8 | | -1 | |  |  |
| RN12 | NGOM | M | 525.2 | 94 | 9/3/11-12/7/11* (95) | | 0.229 | 32.2 | | 36 | | -52 | |  |  |
| RN13 | NGOM | M | 495 | 82 | 9/12/11-12/7/11* (86) | | 0.351 | 13.9 | | 15.2 | | -68 | |  |  |
| *tracked beyond 11/30/11 at same F location | | | | | |  | | |  | |  | |  | |  |
|  | | | | | | | | | | | | | | |  |

**Table S4.** Foraging site characterizations for Kemp's ridley turtles satellite tracked between 1998-2011. F1 was the first foraging period prior to the ‘final’ foraging area, F2 was the foraging period prior to F1, and F3 was the foraging period prior to F2. NA = not available

| Turtle ID | Total no. days in foraging mode (F+F1+F2+F3) | No. mean daily locations at F1 | Dates at F1 (No. days) | Size of 50% KDE at F1 (km^2^) | Bandwidth (h_cv_) | Bathymetry (m) | Distance to nearest shore (km) | Distance to mainland (km) |
| --- | --- | --- | --- | --- | --- | --- | --- | --- |
| 22 | 119 | 35 | 8/3/00-9/25-00 (53) | 1495.3 | 0.4104 | -19 | 28.3 | 34.1 |
| 54 | 249 | 60 | 9/18/04-12/23/04 (96) | 1781.7 | 0.31601 | -16 | 47.1 | 57.4 |
| 21 | 75 | 54 | 6/30/04-9/13/04 (75) | 601.0 | 0.3216 | -20 | 27.4 | 35.4 |
| 12 | 208 | 169 | 6/8/06-1/2/07 (207) | 557.4 | 0.13322 | -13 | 28.1 | 37.9 |
| 125 | 104 | 20 | 6/16/10-7/5/10 (19) | 596.9 | 0.62214 | -18 | 58.1 | 59.2 |
| 319 | 120 | NA | NA | NA | NA | NA | NA | NA |
| 164 | 79 | 78 | 5/27/11-8/13/11 (78) | 2785.0 | 0.14652 | -20 | 47.8 | 48.2 |
| RN13 | 126 | 20 | 8/21/11-9/9/11 (19) | 1386.0 | 0.98904 | -37 | 31.6 | 31.4 |
|  |  |  |  |  |  |  |  |  |
| Turtle ID | Total no. days in foraging mode (F+F1+F2+F3) | No. mean daily locations at F2 | Dates at F2 (No. days) | Size of 50% KDE at F2 (km^2^) | Bandwidth (h_cv_) | Bathymetry (m) | Distance to nearest shore (km) | Distance to mainland (km) |
| 22 | 119 | 56 | 4/25/00-6/30/00 (66) | 3511.0 | 0.26483 | -23 | 61.7 | 63.4 |
| 54 | 249 | 47 | 7/4/04-9/9/04 (67) | 1486.7 | 0.43178 | -17 | 48.5 | 49.2 |
| 21 | 75 | NA | NA | NA | NA | NA | NA | NA |
| 12 | 208 | NA | NA | NA | NA | NA | NA | NA |
| 125 | 104 | NA | NA | NA | NA | NA | NA | NA |
| 319 | 120 | 97 | 6/6/11-9/11/11 (97) | 1184.4 | 0.45544 | -19 | 28.3 | 34.6 |
| 164 | 79 | NA | NA | NA | NA | NA | NA | NA |
| RN13 | 126 | 22 | 7/14/11-8/4/11 (21) | 774.1 | 0.39682 | -15 | 45.1 | 55.7 |
|  |  |  |  |  |  |  |  |  |
| Turtle ID | Total no. days in foraging mode (F+F1+F2+F3) | No. mean daily locations at F3 | Dates at F3 (No. days) | Size of 50% KDE at F3 (km^2^) | Bandwidth (h_cv_) | Bathymetry (m) | Distance to nearest shore (km) | Distance to mainland (km) |
| 319 | 120 | 22 | 4/27/11-5/20/11 (23) | 1413.5 | 0.21043 | -19 | 26.1 | 26.6 |
|  |  |  |  |  |  |  |  |  |

Table S5. Satellite-tracked Kemp’s ridley foraging site characteristics for foraging periods that failed site-fidelity tests.

| **Turtle ID** | **Foraging Area** | **No. mean daily locations** | **Size of 50% KDE (km^2^)** | **Dates (No. days)** | **Bandwidth (h_cv_)** | **Site Fidelity** |
| --- | --- | --- | --- | --- | --- | --- |
| 120 | F1 | 28 | 1870.1 | 6/9/10-7/10/10 (31) | 0.286 | p > 76.2 |
| 54 | F | 61 | 1452.4 | 1/3/05-3/30/05 (86) | 0.158 | p > 35.6 |
| 12 | F | 49 | 75.5 | 1/8/07-3/26/07 (77) | 0.067 | p > 79.2 |
| RN07 | F | 43 | 2960.5 | 8/8/11-12/6/11 (120) | 0.167 | p > 22.8 |
| RN08 | F1 | 34 | 923.0 | 7/22/11-10/29/11 (99) | 0.272 | p > 83.2 |
| RN10 | F | 30 | 1308.4 | 9/11/11-12/7/11 (87) | 0.698 | p > 93.1 |
| RN12 | F1 | 31 | 1108.8 | 8/1/11-8/31/11 (30) | 0.243 | p > 90.1 |
| RN15 | F | 72 | 83.1 | 7/6/11-9/17/11 (73) | 0.053 | p > 48.5 |
| 319 | F1 | 24 | 294.6 | 9/14/11-10/7/11 (23) | 0.294 | p > 64.4 |
| 145 | F1 | 56 | 439.8 | 6/3/11-7/30/11 (57) | 0.125 | p > 45.5 |
| 326 | F | 29 | 1189.7 | 6/25/11-9/17/11 (84) | 0.151 | p > 91.1 |

| **Table S6.** Foraging habitat characterization for Kemp's ridley turtles satellite tracked between 1998 and 2011. Values given are the net primary productivity (NPP) and sea surface temperature (SST) for *N* = 30 kernel density estimations (KDEs) calculated for Kemp's ridleys. F = final foraging area for an individual, F1 = foraging area used immediately prior to F, F2 = foraging area used immediately prior to F1, and F3 = foraging area immediately prior to F2. NA = not available | | | | | |
| --- | --- | --- | --- | --- | --- |
|  |  |  |  |  |  |
|  | NPP (mg C/m^2^/day | Mean | SD | Minimum | Maximum |
|  | All F | 3817.9 | 1175.7 | 734.2 | 5463.96 |
|  | F | 3913.3 | 1394.3 | 734.2 | 5463.96 |
|  | F1 | 3891.0 | 635.9 | 3378.7 | 5188.5 |
|  | F2 | 3232.0 | 1022.9 | 1886.0 | 4140.9 |
|  | F3 | 2789.1 | NA | 2789.1 | 2789.1 |
|  |  |  |  |  |  |
|  |  |  |  |  |  |
|  | SST (°C) | Mean | SD | Minimum | Maximum |
|  | All F | 25.1 | 0.9 | 24.1 | 27.6 |
|  | F | 25.5 | 1.0 | 24.2 | 27.6 |
|  | F1 | 24.6 | 0.3 | 24.2 | 24.9 |
|  | F2 | 24.5 | 0.3 | 24.1 | 24.8 |
|  | F3 | 24.8 | NA | 24.8 | 24.8 |
